# Inside the Borate Anomaly: Leveraging a Predictive Modelling Approach to Navigate Complex Composition–Structure–Property Relationships in Oxyhalide Borate Glasses

**DOI:** 10.3390/ma17092073

**Published:** 2024-04-28

**Authors:** Brenna Kettlewell, Daniel Boyd

**Affiliations:** 1 School of Biomedical Engineering, Dalhousie University, Halifax, NS B3H 4R2, Canada; brenna.kettlewell@dal.ca; 2 Department of Applied Oral Sciences, Faculty of Dentistry, Dalhousie University, 5981 University Avenue, P.O. Box 15000, Halifax, NS B3H 4R2, Canada

**Keywords:** borate glass, design of mixtures, borate anomaly, optimization, predictive modelling

## Abstract

This study employs a systematic and predictive modelling approach to investigate the structure and properties of multi-component borate glasses. In particular, this work is focused on understanding the individual and interaction effects of multiple constituents on several material properties. By leveraging advanced modeling techniques, this work examines how the inclusion and variation of B_2_O_3_, CaF_2_, TiO_2_, ZnO, and Na_2_CO_3_ influence the glass network, with particular attention to modifier fractions ≥ 30 mol%. This research addresses the gap in knowledge regarding the complex behavior of borate glasses in this high modifier fraction range, known as the borate anomaly, where prediction of glass structure and properties becomes particularly challenging. The use of a design of mixtures (DoM) approach facilitated the generation of polynomial equations indicating the influence of mixture components on various responses, enabling the prediction and optimization of glass properties over broad compositional ranges despite being within the anomalous region. This methodical approach not only advances our understanding of borate glass systems but also underscores the importance of predictive modelling in the accelerated design and development of novel glass materials for diverse applications.

## 1. Introduction

Relative to traditional tissue engineering constructs, bioactive glasses are structurally simple, yet functionally complex materials capable of promoting a range of desirable material and host responses via the controlled release of multiple therapeutic inorganic ions (TII) [1,2]. The localized delivery of these bioactive ions offers significant advantages over other chemical and biochemical compounds (e.g., carbohydrates, lipids, proteins, and nucleic acids) for drug delivery. These advantages include, but are not limited to, (i) high thermal stability and thus easier manufacturing, processing, and storage, (ii) the ability to interact synergistically with other ions, (iii) reduced cost, and (iv) the elimination of additional drug loading steps as the therapeutic ions are directly incorporated into the synthesis of the glass itself [3,4]. Similar to typical organic drug biomolecules, such as growth factors, TIIs have been demonstrated to alter cellular functions and metabolism as enzyme cofactors and/or by activating ion channels or secondary signaling [3,5]. The controlled release of TIIs is dependent on the composition of the glass and how each constituent alters the structural characteristics of the network. Therefore, to understand how TIIs can be harnessed to modulate various biological responses (e.g., antibacterial, anti-inflammatory, osteoinductive, and angiogenic processes), one must first understand the physical, chemical, and structural properties of a given glass system.

Within the vast landscape of glass compositions capable of eliciting desired material and host responses, it is noteworthy that a significant focus in the investigation of bioactive glasses centers around silicate glass networks [6]. Despite their prominence, it is important to acknowledge that these glass systems present challenges in some indications, such as slow and incomplete degradation. This is attributed to the low solubility of silica and the formation of a silica-rich gel layer on the glass surface, which, in turn, restricts the diffusion of ions into the solution [7,8,9]. In the context of bone tissue engineering, for example, this translates into slow and incomplete conversion to hydroxyapatite (HA), which results in decreased rates of bone formation, longer healing times, and residual materials at the implant site [9,10]. As a result of such limitations, there exists opportunities to discover and explore a broader spectrum of glass compositions with distinct degradation profiles and payloads of TIIs. Contrary to their silicate counterparts, and of increasing interest in the literature, borate glasses undergo complete degradation, a process that can be adjusted through the selection of specific TIIs, characterized as either network modifying, network forming, or intermediate elements, and their inclusion into the molecular network of the glass [11]. Consequently, borate glasses are gaining increasing interest in biomedical applications for their ability to act as fully resorbable carrier systems for the controlled and localized delivery of TIIs [12,13,14].

Unlike the properties of silicate glasses, which vary monotonically with the network-modifying fraction of oxides contained within the glass structure, borate glass properties exhibit extrema in response to specific quantities of network modifier(s) and the presence of certain basic structural and superstructural units [11]. For instance, upon the addition of a network modifier to the borate glass network, the trigonal BØ_3_ structural units convert to tetrahedral BØ_4_^−^ units. This conversion takes place until an added modifier fraction of approximately 30 mol% has been reached, whereafter, further addition of modifier leads to the formation of non-bridging oxygens and the subsequent decrease in BØ_4_^−^ units [11,15]. An increased number of four-coordinated boron structural units increases the connectivity of the glass network, thus providing enhanced hydrolytic stability and resistance to degradation. In contrast, the formation of non-bridging oxygens leads to a more disrupted glass network and, hence, a more reactive material. Therefore, the type and fraction of network-modifying elements included in the network can be tailored to modulate the structure and dissolution of borate glasses, providing improved control and predictability over a range of material and host responses.

The non-linear structural behavior exhibited by borate glasses is referred to as the borate anomaly [11,16]. This behavior is the basis upon which glasses containing a network modifier fraction greater than 30 mol% are notoriously difficult to predict and thus vastly understudied [6,11]. Furthermore, the behavior of borate glass systems has primarily been reported for binary glass substitutions of alkali or alkaline earth network modifiers [11]. Hence, the properties of ternary and quaternary borate glass systems are more poorly understood, while those of greater complexity, comprising multiple cations and anions with variable valences and cationic field strengths, are even less predictable. 

As such, the ongoing investigation of borate glass systems is currently faced with exciting opportunities for growth. The potential for streamlined development of these materials, especially those containing more than two components, substitutions from groups beyond alkali and alkaline earth metals, and modifier fractions exceeding 30 mol%, is substantial. However, as with other advanced material technologies, the accelerated translation of these materials towards clinical use has been somewhat hindered by a current lack of comprehensive knowledge surrounding their structure and properties [17,18,19]. This limited understanding is associated with the use of traditional trial-and-error style approaches and/or one-variable-at-a-time (OVAT) methodologies in the study of glass materials. While these approaches have played a significant role in the design and development of novel glass materials, the sequential adjustments of individual variables characteristic of these methodologies can be extremely time-consuming, resource intensive, and impractical for investigating extensive combinatorial palettes of glass chemistries. Specifically, trial-and-error or OVAT approaches may inadvertently overlook or underestimate the complexity of multi-component glasses and the likelihood for elements to interact with each other, forming synergistic relationships within the network that may influence material and host responses. Hence, this complicates our ability to predict the individual *and* interaction effects of glass constituents, especially in systems comprising multiple cations and anions with variable valences and cationic field strengths. Without the ability to gain insights into these complex relationships, traditional methodologies may not efficiently navigate the multidimensional space of compositional factors, thus precluding the simultaneous optimization of multiple variables. In light of these considerations, there is a clear imperative for more sophisticated and comprehensive approaches to ensure scientific rigor, accuracy, and sustainability in the design of glass materials [20,21,22].

Further investigation of borate glass materials is necessary to support their accelerated discovery, design, and deployment across a wide range of indications, including biomedical, nuclear, and industrial applications. Given the complex nature of these systems, quantifying and predicting the composition–structure–property relationships within these materials requires comprehensive and systematic evaluation. In this regard, the Materials Genome Initiative (MGI) advanced a new paradigm for accelerated materials innovation [19]. The approach of the MGI encourages the combination of experimentation, theory, and computation to permit the high-throughput screening, prediction, and optimization of new materials and promotes the collaboration of researchers based on shared access to numerous materials datasets [19,23]. One systematic approach that conforms to the objectives of the MGI and supports the accurate prediction of materials properties across broad compositional ranges is the design of mixtures (DoM) approach. This approach simultaneously integrates experimental methods and advanced modelling to generate statistically driven polynomial equations that indicate the relative influences of mixture components, both individually and interactively, on a given response. Using surface regression methodologies, this approach permits the prediction and optimization of materials for a wide variety of desirable properties, which, unlike traditional trial-and-error style approaches, elucidates a more deliberate and streamlined design of materials [24,25,26,27,28]. Accordingly, the primary objective of the current study is to employ a DoM approach to predict the individual and interaction effects of multiple constituents on the physical and chemical properties of multi-component, degradable borate glasses with modifier fractions ≥ 30 mol%, a compositional region that is poorly understood and difficult to predict.

## 2. Material and Methods

### 2.1. Material Design and Synthesis

The compositions for each glass were established using Design-Expert Software (Version 13). A DoM I-optimal quadratic model was utilized based on five components (B_2_O_3_, CaF_2_, TiO_2_, ZnO, Na_2_CO_3_). Specifically, the I-optimal algorithm was chosen to accommodate this complex mixture, comprising multiple components with varying compositional ranges. Furthermore, this algorithm chooses formulations (i.e., runs) that minimize the integral of prediction variance across the design space, making it the recommended algorithm for optimization of the materials. A quadratic model was necessary to gain insight into not only the individual effects of each component (as provided with a linear model), but also the second-order interaction effects. Various test melts were conducted to confirm the compositional constraints of each constituent (Table 1). Based on these constraints, the model yielded a total of 23 randomized glass formulations for investigation (Table 2), consisting of 15 model points, 4 lack of fit points, and 4 replicates. Replicate compositions were included to verify reproducibility in glass synthesis and characterization processes and to increase the predictive power of the model. Using standard analysis of variance (ANOVA), a quadratic Scheffe polynomial equation was fitted to each measured response based on the following equation:

Y = β_1_A + β_2_B + β_3_C + β_4_D + β_5_E + β_12_AB + β_13_AC + β_14_AD + β_15_AE + β_23_BC + β_24_BD + β_25_BE + β_34_CD + β_35_CE + β_45_DE

where A to E represent the compositional factors, β_1–5_ coefficients represent the effect of the individual compositional factors A to E, and β_12–45_ are the coefficients of regression, which represent the effects of the interactions between the compositional factors A to E [29]. This response surface regression model approach was used to quantitatively determine the relative impacts of each glass constituent, both individually and interactively, on variable material properties, such as density, T_g_, and fraction of B3 and B4 structural units.

Glasses were synthesized via the traditional melt quenching technique. Succinctly, analytical-grade boric anhydride, calcium fluoride, titanium (IV) oxide, zinc oxide, and sodium carbonate reagents (Sigma-Aldrich, Burlington, MA, USA) were weighed out in accordance with compositions listed in Table 2 and homogenized for 1 h prior to being transferred to 50 mL Pt-Rh crucibles (XRF Scientific, Montreal, QC, Canada) for melting [30]. The crucible was placed in a high-temperature box furnace (Carbolite, RHF 16/3) and followed a two-step thermal process; materials were heated from room temperature to 850 °C (10 °C/min) and held for 2 h for calcination, then ramped from 850 °C to 1200 °C (10 °C/min) and held for 1 h for melting. Each melt was quenched using stainless steel plates, and the resulting frit was ground and sieved (ASTM E-11 [31] compliant sieves) to obtain particles in size ranges of <90 μm, 90–710 μm, and > 710 μm diameter (Superla Sieve™, Newark Wire Cloth Company, Clifton, NJ, USA). Glasses were stored in desiccated conditions until the time of subsequent analysis.

### 2.2. Particle Size Analysis

A Malvern Mastersizer 3000 laser diffraction particle size analyzer was used as per the manufacturer’s instructions. Samples of each glass, sieved to 90–710 µm, were suspended in deionized water to obtain an obscuration value for the suspension between 5 and 8%. The glass suspension was then measured using both a blue (λ = 470 nm) and a red (λ = 632.8 nm) laser. Each glass suspension was measured five times (n = 5), and particle size distribution data were reported as the mean diameter Dx90, Dx50, and Dx10 (particle diameters at 90%, 50%, and 10% cumulative size, respectively).

### 2.3. X-ray Diffraction

X-ray diffraction analysis was performed using a Bruker D2 phaser (Bruker, Billerica, MA, USA) equipped with a Lynx-Eye 1D detector (linear array). Powder specimens of each composition were loaded into a PMMA sample holder and analyzed in the region between 10° ≤ 2θ ≤ 60° with a step size of 2θ = 0.03 and a step time of 2 s. XRD spectra were analyzed using Bruker Diffrac.Eva (https://www.bruker.com/en/products-and-solutions/diffractometers-and-x-ray-microscopes/x-ray-diffractometers/diffrac-suite-software/diffrac-eva.html) to calculate the relative fraction of crystalline material in each sample by evaluating the amorphous baseline to crystalline peak separation. Where applicable, Diffrac.Eva software was used to identify crystalline phases through peak matching against reference databases. 

### 2.4. Density

An AccuPyc 1340 helium pycnometer (Micromeritics, Norcross, GA, USA) equipped with a 1 cm^3^ insert chamber was used to determine the density of each glass composition as per manufacturer instructions [32]. Briefly, 0.5–0.6 g of glass powder was used for each measurement, and each measurement consisted of 10 fill and purge cycles (run in triplicate). The results were reported as the average ± standard deviation (SD) of three replicate measurements. 

### 2.5. Differential Scanning Calorimetry

A simultaneous thermal analysis Luxx 409 PC STA with Auto-Sampler (Netzsch-Geratebau-GMBH, Exton, PA, USA) was used to analyze each glass composition. Approximately 20–30 mg of glass powder was weighed out, placed into Pt-Rh crucibles, and heated at 10 °C/min from 30 to 1000 °C (Standard Test Method for Assignment of the Glass Transition Temperatures by Differential Scanning Calorimetry, ASTM E1356 [33]). Proteus Thermal Analysis software (Version 8.0.2) was used to determine the extrapolated glass transition temperature (T_g_) (inflection). 

### 2.6. ^11^B MAS NMR

^11^B magic angle spinning (MAS) NMR spectra were acquired using a 16.4 T Bruker Avance NMR spectrometer (^11^B Larmor frequency = 224.67 MHz) equipped with a 2.5 mm HX probe head operating in single resonance mode. ^11^B parameters were calibrated on solid NaBH_4_, which was also used as a secondary chemical shift reference (−42.1 ppm relative to BF_3_·Et_2_O). All samples were spun at 20 kHz MAS frequency, producing 64 scans. A single 0.63 µs pulse was used for all experiments, corresponding to a pulse angle of roughly 15° in a nearby cubic environment of NaBH_4_. Spin lattice relaxation times were determined separately for the B3 and B4 groups using a saturation recovery sequence and were found to be around 6–9 s. The pulse repetition times were chosen to be 5 s. The boron background was removed by subtracting the spectrum of the used empty rotor from the sample spectra. After baseline correction with a spline function, the peak heights, widths, and integral values were determined for the B3 and B4 groups using XWinNMR’s native tools.

### 2.7. Optimization and Validation

A response surface optimization study using Design-Expert Software (Version 13) was conducted to predict two new desirable glass compositions tailored to meet a set of optimization criteria (listed in Table 3). The optimization criteria for the two formulations (denoted as optimized glass A and B) were chosen to yield solutions with distinct compositions. This design permits a comprehensive investigation of model predictability across two glass systems exhibiting variable structure and properties. For instance, it was preferred that optimized glass A feature a B_2_O_3_ content at the upper end of the compositional constraints (70 mol%), thus representing a composition on the upper limit of the anomalous region (i.e., modifier fraction = 30 mol%), as defined by the binary borate glass literature [11,15]. In contrast, optimized glass B was preferred to have a B_2_O_3_ content within the specified compositional range (45 to 70 mol%), thus representing a composition within the anomalous region. Design-Expert software systematically generated numerous potential solutions based on the optimization criteria selected. 

The two optimal glass formulations were chosen based on the desirability score assigned to each solution. The desirability score serves as an objective function, ranging from zero (outside of the limits) to one (at the goal), and is contingent on how closely the lower and upper limits align with the actual optimum. The numerical optimization process identifies a point that maximizes the desirability function. Therefore, at both optimized settings, the solution with the highest desirability score was selected as the preferred candidate. 

The two chosen optimized formulations were synthesized using the same parameters as the initial 23 glasses. Subsequently, density, DSC, and ^11^B MAS NMR analyses were repeated on the optimized glasses using the same parameters as the initial 23 glasses. Furthermore, XRD analysis was conducted on the optimized glasses to verify the absence of identifiable crystalline species, with a doubled step time to account for the use of a low background sample holder. The actual experimental values were compared to the predicted values of the model using a two-sided confidence interval of 95% to validate the predictive power of the model. If actual values fell within the 95% prediction intervals, this indicated that the model predicted accurately at those optimized settings. 

## 3. Results

### 3.1. Glass Synthesis

All compositions were successfully synthesized and formed flowable melts capable of being poured and quenched. The quenched glasses varied in opacity. Compositions #7, 10, and 19 were almost entirely white opaque materials upon quenching, which were later verified as partially crystalline materials via XRD analysis, exhibiting % crystallinities of 12%, 17%, and 15%, respectively. 

### 3.2. Particle Size Analysis

Each glass was processed to meet the particle size distribution of 90–710 µm. The Dx10, Dx50, and Dx90 values for each glass composition were measured (Table 4) to confirm repeatability and reproducibility in the glass processing. 

### 3.3. Density

**Figure 1:**
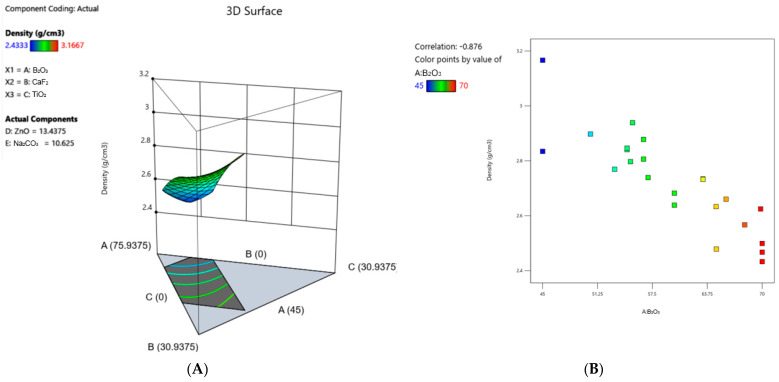
(**A**) 3D surface plot representing the density response across the compositional ranges of each constituent in the series. (**B**) Correlation graph of density vs. B_2_O_3_ content.

Density values ranged from 2.43 to 3.17 g/cm^2^ (Figure 1A, Table 4). A quadratic model with statistical significance, as represented in Table 5, was developed to understand the relative influence of glass constituents, both individually and interactively, on glass density. Examination of the coefficients relating to the model suggests that factors contributing to an increase in the density response, ranked in order of magnitude, are B_2_O_3_*Na_2_CO_3_ > TiO_2_ > ZnO > CaF_2_*Na_2_CO_3_ > CaF_2_ > ZnO*Na_2_CO_3_ > B_2_O_3_ > TiO_2_*Na_2_CO_3_ > B_2_O_3_*CaF_2_. Factors that contribute to a decrease in density, ranked in order of magnitude, are TiO_2_*ZnO > B_2_O_3_*TiO_2_ > Na_2_CO_3_ > CaF_2_*TiO_2_ > CaF_2_*ZnO > B_2_O_3_*ZnO. A 3D surface plot (Figure 1A) representing the density response with respect to the variable concentrations of each glass constituent is also shown. The strongest correlation observed from the density data was an increase in density with decreasing B_2_O_3_ content (Figure 1B). Full model details, including coded coefficients and ANOVA outputs (R^2^, adjusted R^2^, predicted R^2^, C.V. (%), and adequate precision), are provided in Table 5. The predicted R^2^ is within reasonable agreement with the adjusted R^2^ (i.e., the difference between the two values is less than 0.2), and the adequate precision (i.e., signal-to-noise ratio) is greater than 4 for every response, indicating the predictive power of the models.

### 3.4. X-ray Diffraction (XRD) 

**Figure 2:**
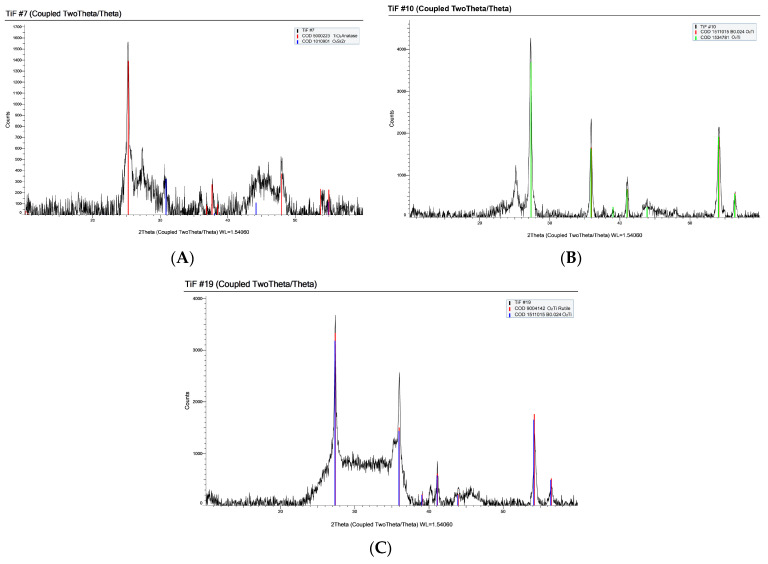
XRD crystal peak matching of samples (**A**) #7, (**B**) #10, and (**C**) #19.

**Figure 3:**
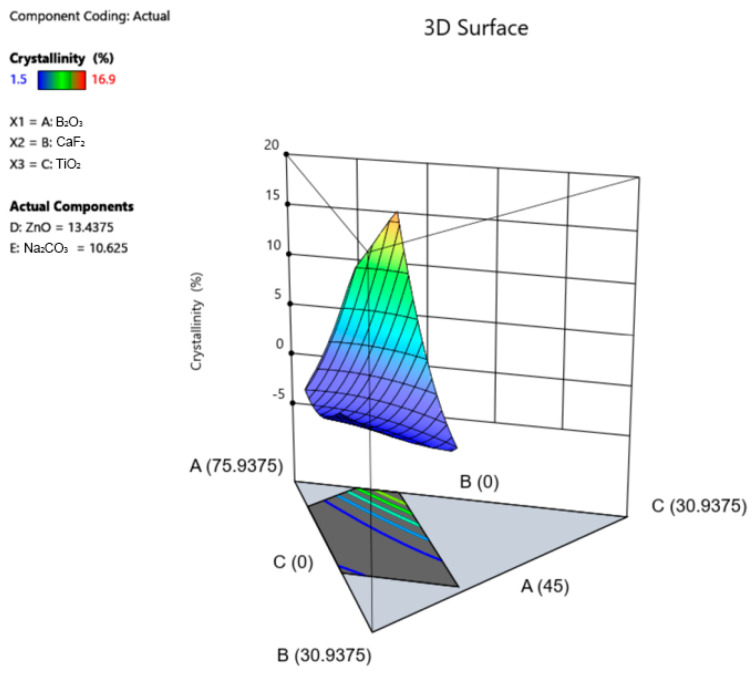
3D surface plot representing the % crystallinity response across the compositional ranges of each constituent in the series.

**Figure 4:**
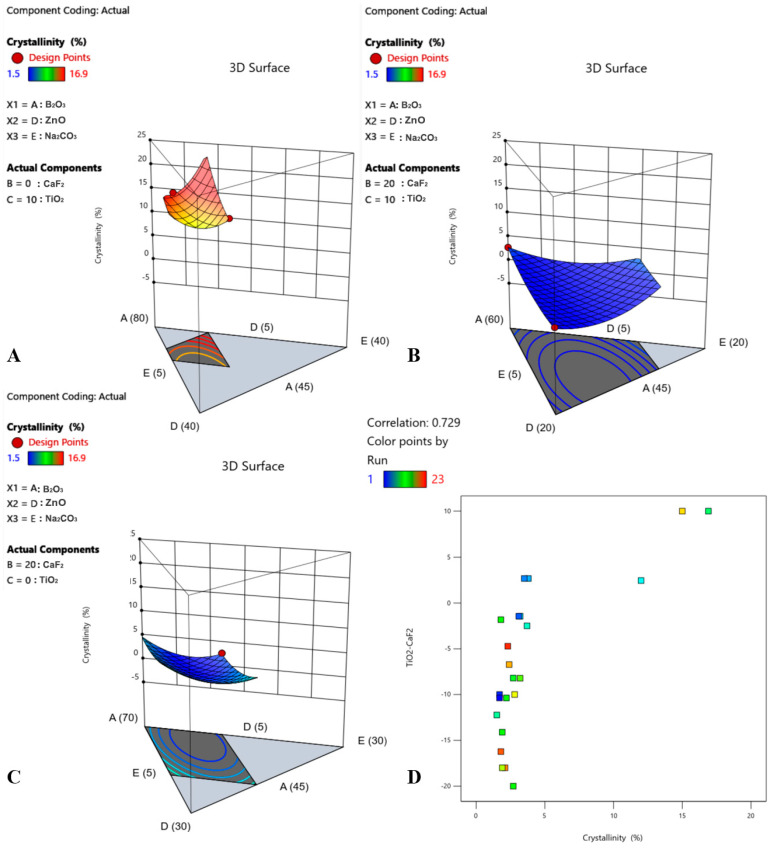
3D surface plots representing the % crystallinity response for three distinct scenarios in which the concentrations of CaF_2_ and TiO_2_ vary but the other components remain constant ((**A**) Minimum CaF_2_ and maximum TiO_2_; (**B**) maximum CaF_2_ and maximum TiO_2_; (**C**) maximum CaF_2_ and minimum TiO_2_). (**D**) Correlation graph of crystallinity vs. TiO_2_-CaF_2_.

XRD analysis revealed varying degrees of crystallinity, ranging from 1.5% to 16.9% (Table 4). All glasses were free of identifiable crystalline species except samples #7, 10, and 19. The XRD spectra of these three samples showed sharp, visible peaks (Figure 2). Peak matching revealed that these crystalline peaks were characteristic of various titanium crystals (Table 6). Crystallinity data were used to derive a quadratic model with statistical significance (Figure 3, Table 5). The factors that contribute to an increase in crystallinity, ranked in order of magnitude, are TiO_2_ > Na_2_CO_3_ > CaF_2_ > ZnO > B_2_O_3_. Factors that contribute to a decrease in crystallinity, ranked in order of magnitude, are CaF_2_*TiO_2_ > CaF_2_*Na_2_CO_3_ > ZnO*Na_2_CO_3_ > TiO_2_*Na_2_CO_3_ > B_2_O_3_*Na_2_CO_3_ > TiO_2_*ZnO > B_2_O_3_*CaF_2_ > CaF_2_*ZnO > B_2_O_3_*ZnO > B_2_O_3_*TiO_2_. An additional finding with respect to the crystallinity of the glasses was an apparent effect between the TiO_2_:CaF_2_ concentration and crystallinity. To examine this effect, components B: CaF_2_ and C: TiO_2_ were removed from the axes of the 3D surface plot for crystallinity to permit visualization of the response surface upon adjusting these two constituents (Figure 4A–C). As shown in Figure 4A–C, the crystallinity of the glasses is at its maximum when the CaF_2_ content is minimized (0 mol%) and the TiO_2_ content is maximized (10 mol%). In contrast, the crystallinity is low when the CaF_2_ content is maximized (20 mol%) and the TiO_2_ content is maximized, or, similarly, if the CaF_2_ content is maximized and the TiO_2_ content is minimized (0 mol%). In addition, the difference between TiO_2_ content and CaF_2_ content was modelled to investigate whether there was a correlation between crystallinity and TiO_2_–CaF_2_ (Figure 4D).

### 3.5. 11B MAS NMR

**Figure 5:**
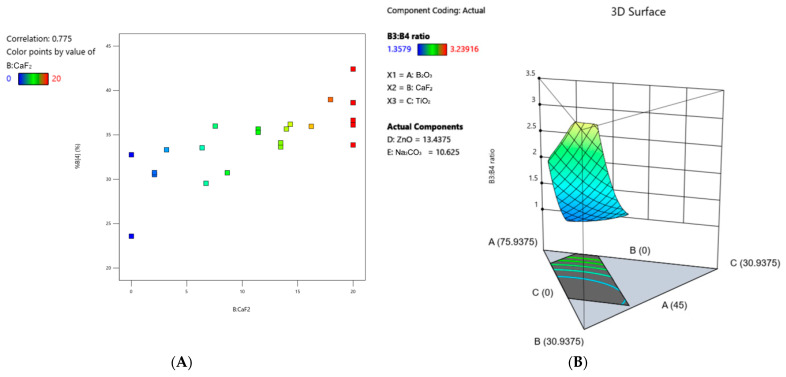

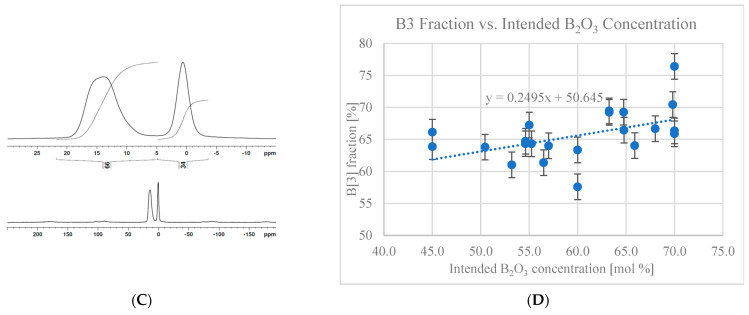
(**A**) 3D surface plot depicting the ratio of B3:B4 structural units relative to varying amounts of each constituent in the series. (**B**) Correlations graphs of %B4 vs. CaF_2_ content. (**C**) Representative ^11^B MAS NMR spectra for the glasses under investigation (sample #1). (**D**) Experimentally obtained concentration of B3 versus intended B_2_O_3_ concentration for each glass in the series.

A representative ^11^B MAS NMR line spectra is provided in Figure 5C. The peaks represent the B3 (12–20 ppm) and B4 ((−3)–5 ppm) units in the sample. The ^11^B MAS NMR data for %B3 were fitted to a quadratic model with statistical significance (Table 5). The %B3 values ranged from 57.6 to 76.4% (Table 4). The strongest correlation observed from the ^11^B MAS NMR data was an increase in the B4 fractions and a decrease in the B3 fractions with increasing quantities of CaF_2_ (Figure 5A). Analysis of the coefficients derived from the coded equation for this response indicate that the glass constituents that contribute to an increase in %B3, ranked in order of magnitude, are B_2_O_3_ > ZnO > TiO_2_ > CaF_2_ > Na_2_CO_3_ > ZnO*Na_2_CO_3_ > CaF_2_*Na_2_CO_3_. The glass constituents that contributed to a decrease in %B3, ranked in order of magnitude, are B_2_O_3_*CaF_2_ > B_2_O_3_*Na_2_CO_3_ > B_2_O_3_*ZnO > TiO_2_*ZnO > B_2_O_3_*TiO_2_ > CaF_2_*TiO_2_ > CaF_2_*ZnO > TiO_2_*Na_2_CO_3_. 

Similarly, the data for %B4 were fitted to a quadratic model with statistical significance (Table 5). The %B4 values ranged from 23.6 to 42.4% (Table 4). The factors that contribute to an increase in %B4, ranked in order of magnitude, are B_2_O_3_*CaF_2_ > B_2_O_3_*Na_2_CO_3_ > Na_2_CO_3_ > B_2_O_3_*ZnO > CaF_2_ > TiO_2_ > ZnO > TiO_2_*ZnO > B_2_O_3_*TiO_2_ > CaF_2_*TiO_2_ > B_2_O_3_ > CaF_2_*ZnO > TiO_2_*Na_2_CO_3_. The factors that contribute to a decrease in %B4, ranked in order of magnitude, are ZnO*Na_2_CO_3_ > CaF_2_*Na_2_CO_3_. 

Lastly, analysis of the B3:B4 ratio (Figure 5B) produced a statistically significant quadratic model (Table 5), with values ranging from 1.358 to 3.24 (Table 4). The factors that contributed to an increase in B3:B4, ranked in order of magnitude, are TiO_2_ > B_2_O_3_ > ZnO > CaF_2_ > CaF_2_*Na_2_CO_3_ > ZnO*Na_2_CO_3_ > Na_2_CO_3_. The factors that contributed to a decrease in B3:B4, ranked in order of magnitude, are CaF_2_*TiO_2_ > B_2_O_3_*CaF_2_ > TiO_2_*Na_2_CO_3_ > TiO_2_*ZnO > B_2_O_3_*Na_2_CO_3_ > B_2_O_3_*TiO_2_ > B_2_O_3_*ZnO > CaF_2_*ZnO. 

**Figure 6:**
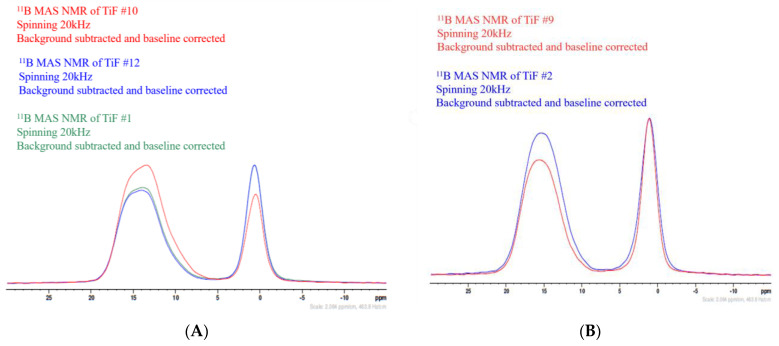
Overlay plots of glass samples having the same concentration of B_2_O_3_, but varying concentrations of additive elements. (**A**) Overlay plot of sample #1, 12, 10, all having 70 mol% B_2_O_3_ (note: samples #1 and 12 are replicate compositions). (**B**) Overlay plot of sample #9 and 2, both having 45 mol% B_2_O_3_.

Additionally, the experimental fraction of trigonally coordinated boron was plotted against the intended B_2_O_3_ concentration in the glasses (Figure 5D). The 2% error bars (vertical axis) account for potential variations in the NMR data when determining the relative fraction of B3. As shown in Figure 5D, the data points are very scattered, and therefore, the correlation between B_2_O_3_ content and boron coordination is somewhat ambiguous. To investigate this scattering further, NMR spectra for samples with identical B_2_O_3_ concentrations but varying concentrations of additive components were overlayed to compare the B3 and B4 sites (e.g., position and shape) and investigate the impact of varying modifiers on glasses with constant B_2_O_3_ content. Two representative overlay plots are shown in Figure 6A,B, wherein the B3:B4 ratios clearly varied across samples. Although some variation between samples was expected due to differences in the modifying elements and their concentrations, these deviations warranted further investigation. 

**Figure 7:**
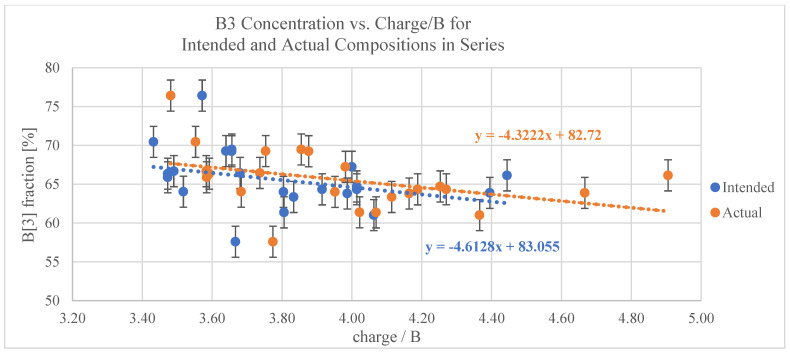
Fraction of B3 versus the overall cation charge per boron atom based on the intended composition of each glass (blue) and the actual, experimental composition of each glass (orange).

Because these glasses are highly complex, consisting of several different cations as well as oxygen and a fluoride, a typical B3 to O:B correlation could not be used. Alternatively, the B3 content was plotted against the overall cation charge per boron atom based on the intended and actual compositions (Figure 7). For pure B_2_O_3_, the charge per boron is 3, and one would expect to see 100%B3. Similarly, for tetrahedrally coordinated boron, one would see 0%B3. Based on this, a slope of −100%/charge is expected. However, as shown in Figure 7, the slopes correlating with the raw NMR data were −4.6 and −4.3, respectively. 

### 3.6. Differential Scanning Calorimetry

**Figure 8:**
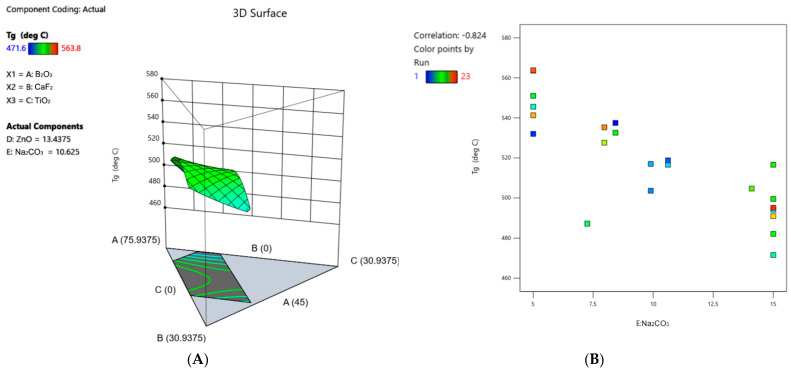
(**A**) 3D surface plot representing the T_g_ response across the compositional ranges of each constituent in the series. (**B**) Correlation graph of T_g_ vs. Na_2_CO_3_ content.

T_g_ values ranged from 471.6 °C to 563.8 °C (Table 4). These values were fitted to a quadratic model with statistical significance (Figure 8A, Table 5). Analysis of the coefficients derived from the coded equation describing this model indicates that the glass constituents contributing to an increase in T_g_, ranked in order of magnitude, are Na_2_CO_3_ > TiO_2_ > ZnO > B_2_O_3_*CaF_2_ > CaF_2_ > B_2_O_3_ > CaF_2_*TiO_2_ > B_2_O_3_*ZnO > TiO_2_*ZnO > CaF_2_*ZnO. Constituents that contribute to a decrease in T_g_, ranked in order of magnitude, are TiO_2_*Na_2_CO_3_ > ZnO*Na_2_CO_3_ > CaF_2_*Na_2_CO_3_ > B_2_O_3_*Na_2_CO_3_ > B_2_O_3_*TiO_2_. The strongest correlation observed from DSC data was an increased T_g_ with decreasing Na_2_CO_3_ content (Figure 8B).

**Figure 9:**
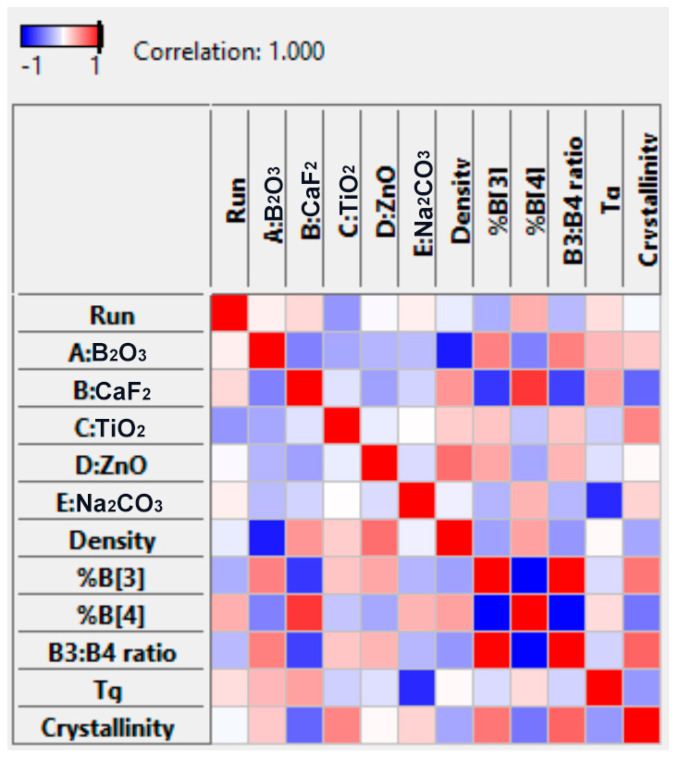
Correlation table indicating correlations that exist between glass constituents and responses. Correlations are represented on a color scale, wherein red indicates a positive correlation and blue indicates a negative correlation.

As shown by the coefficients in the coded equations in Table 5, the individual and interaction terms associated with a given constituent can have opposing impacts. To provide enhanced clarity on the overall impact of a single constituent on a given response, a graphical correlation table is depicted in Figure 9. 

### 3.7. Optimization and Validation

The two optimal glass formulations derived from the optimization criteria outlined in Table 3 are shown in Table 7. The predicted values for density, %B3, %B4, T_g_, as well as desirability scores for each optimized formulation, are also included in Table 7. 

XRD analysis confirmed that both optimized glasses were free from identifiable crystalline species. The actual experimental values derived from density, DSC, and ^11^B MAS NMR analyses of optimized glasses A and B are listed in Table 8 and Table 9, respectively, as well as the predicted values of the model and the 95% prediction interval lower and upper limits. As shown in Table 8 and Table 9, the actual values are within the 95% prediction interval for density and T_g_. However, the results for %B3 and %B4 fall outside the model’s prediction interval, indicating that the %B3 and %B4 models are not predicting as accurately as desired. 

## 4. Discussion

The non-linear behavior exhibited in borate glass systems is referred to as the borate anomaly and is the reason why the structure and properties of borate glasses with modifier fractions greater than 30 mol% are very difficult to predict [11,16]. The difficulty in predicting the composition–structure–property relationships of borate glasses in this high modifier fraction range introduces a significant hurdle in advancing the optimization of glass compositions beyond conventional limits. In essence, addressing the challenges posed by the borate anomaly has the potential to unlock new avenues for innovation in glass science and broaden the scope of materials with enhanced functionalities. 

Recognizing the limitations of traditional trial-and-error or OVAT approaches in understanding the constituent interactions that occur within such complex glass systems has encouraged a shift towards adopting more advanced methodology. As such, a DoM approach was taken for this investigation. This approach stands out for its ability to discern both the individual and interaction effects of each glass constituent on material properties, thus offering a more profound understanding of the convoluted relationships that exist within a glass system. Moreover, it permits rapid screening across broad compositional ranges, thus directly supporting the accelerated design, development, and deployment of novel glass materials. The glass system under investigation in this work was designed (from a compositional standpoint) within an interesting region of the borate anomaly where structural behavior is not well defined [11]. Specifically, these glasses have modifier fractions ranging between 30 and 55 mol%. While this region offers the opportunity to experiment within a wide range of structural constraints, the variability in network properties makes it extremely difficult to predict. This challenge is exacerbated by the inclusion of multiple elements belonging to various groups (i.e., groups 1, 2, 4, 12, 13, 17) and exhibiting variable physical properties. 

As shown in Table 5, the DoM statistical modelling approach has permitted the generation of polynomial equations with coded coefficients that indicate the relative influence of each constituent on variable responses. These equations are dependent on the design constraints listed in Table 1, wherein the concentrations of each constituent (in mol%) are constrained to add to a fixed total (i.e., 100 mol%), meaning that the value of a given constituent is necessarily dependent on the values of the others. These polynomial equations are extremely valuable predictive tools and clearly illustrate that the complex interactions that occur between glass constituents can have massive implications on material structure and properties. To gain insight into whether a given constituent is causing an increase or decrease in a response, the polynomial equation should be used in conjunction with the correlations table (Figure 9) and the 3D surface plots. The correlations table is a direct visualization of the inputs versus the outputs (i.e., data versus data), independent of any model. This table is extremely valuable in identifying any correlations that exist between two properties or between property and composition. Alternatively, the 3D surface plots are a visual representation of a given response relative to the variable concentrations of each constituent in the glass. The 3D surface plots alone demonstrate the non-linearity and unpredictability of relationships within these complex glass systems. This graphical representation not only showcases the complexity of the system but also highlights the utility of predictive modelling approaches. Specifically, these systematic methods simultaneously predict the individual and interaction effects of multiple constituents on material properties over vast compositional ranges, providing a comprehensive understanding that transcends linear interpretations.

As shown in Figure 1B, there was a linear correlation observed between density and B_2_O_3_ content in these glasses, wherein the density increased with decreasing B_2_O_3_ content. This behavior can also be seen in the 3D surface plot representing the density response (Figure 1A), wherein the surface contour is at its lowest point and component A: B_2_O_3_ is at its maximum. This trend may be explained by the increasing fraction of modifying elements that is associated with a decreasing B_2_O_3_ content, as these modifying elements have higher atomic masses compared to boron (e.g., the atomic mass of B is 10.811 g/mol while the atomic masses of Ti, Zn, Ca, and F are 47.867, 65.38, 40.078, and 18.998 g/mol, respectively). Similar findings have been reported in the literature with respect to the effect of ZnO [34] and TiO_2_ [35] on increasing the density of borate glasses. This is further supported by the relative impact of each modifying element on density, as per the coded equation in Table 5, wherein the constituents with larger atomic masses (e.g., TiO_2_ and ZnO) had the greatest individual impacts on increasing density, as would be expected. Furthermore, titanium and zinc are classified as intermediate elements according to Sun and Dietzel’s classifications, meaning they can act as glass network modifiers or glass network formers depending on the glass composition [16]. For instance, titanium has been shown to exist in [TiO_4_], [TiO_5_]^2−^, and [TiO_6_]^2−^ coordination states, wherein it assumes different roles based on its structure and the type of glass network [36]. Specifically in borate glass networks, titanium has been reported to exist as a network former in the TiO_4_ tetrahedral state and a network modifier in the TiO_6_^2−^ octahedral state [26,29,30,37,38,39]. Similarly, in borate glass networks, zinc has been shown to act as a network former in the fourfold coordination state and a network modifier in the sixfold coordination state [40,41,42,43,44,45]. Therefore, it is possible that these constituents increased the density of these glasses by (1) assuming a network-forming role, thus contributing to building the basic framework of the glass, or (2) acting as a network modifier and forming crosslinks between existing borate units, thus increasing the network connectivity of the glass. For instance, in a borate glass network, the addition of TiO_2_ up to 15 mol% was reported to cause a substitution of BO_3_^3−^ units with Ti^4+^ units due to the small ionic radius and large electric charge of Ti^4+^ ions [46]. This resulted in the formation of B–O–Ti bonds rather than B–O–B bonds and was reported to cause increased network connectivity. Alternatively, a study by El-Falaky and Guirguis found that the addition of ZnO up to 35 mol% to a borate glass caused a decrease in tetrahedrally coordinated boron, as ZnO_4_ assumed the role of a network former and gradually replaced borate units. This resulted in an increase in the number of bonds per unit volume, an increased packing density, and a decreased molar volume [44]. These findings reiterate how glass structural behavior varies depending on the number of modifying elements, the properties of these modifying elements, and the concentrations of each element in the network, further emphasizing the importance of understanding the influence that each constituent has on material properties, both individually and interactively.

Another interesting finding observed in this work was the correlation between the appearance of quenched glass samples and their measured % crystallinities. The quenched glass samples varied in appearance, some being completely transparent, and others with varying fractions of white, opaque coloring mixed throughout. XRD analysis confirmed the absence of identifiable crystalline species in all samples, except for samples # 7, 10, and 19. These three samples had the largest fractions of white opaque coloring and had the highest % crystallinities, according to XRD analysis. Peak matching of the XRD spectra pertaining to these three samples revealed the presence of various titanium crystals, such as anatase and rutile, indicating that titanium is likely phase separating into crystalline phases. Similar findings have been reported in the literature with respect to the role of titanium in inducing crystallization in various glasses and glass ceramics [36,38,46,47,48,49,50,51]. In fact, titanium is widely acknowledged as an effective nucleating agent and is believed to act as a catalyst for initiating crystallization centers that induce controlled bulk crystallization and subsequent volume crystallization [38]. Interestingly, as shown in Figure 4, there appeared to be an effect between the CaF_2_/TiO_2_ ratio and the crystallinity of the glasses, wherein samples with the greatest % crystallinities included higher concentrations of TiO_2_ compared to CaF_2_. This effect was further supported by the correlation between crystallinity and TiO_2_–CaF_2_, wherein an increased % crystallinity was correlated with an increased difference between TiO_2_ and CaF_2_. This suggests that the presence of CaF_2_ may have hindered the formation of titanium crystalline phases within the glasses. The potential Ti crystalline phase separation in the glasses under investigation was further evidenced through ^11^B MAS NMR findings.

The ^11^B MAS NMR data in Figure 5D show the percentage of trigonally coordinated boron plotted against the intended B_2_O_3_ content in the glasses. Contrary to what is known in the binary borate glass literature with respect to changes in boron coordination with varying the B_2_O_3_ content, there were no identifiable trends between the B3 fraction and the B_2_O_3_ content for these glasses. Furthermore, the data points in Figure 5D appeared to be very scattered. To investigate the cause of the scattering, the NMR spectra for samples with identical B_2_O_3_ concentrations but varying concentrations of additive components were overlayed to compare the position and shape of the B3 and B4 sites (representative plots shown in Figure 6A,B). These overlay plots, in combination with Figure 5B, demonstrate how, even across samples having identical B_2_O_3_ content, the B3:B4 ratios varied, which can be attributed to the differences in modifier type and concentrations. For instance, these variations may be a result of some of the Ti^4+^ being exchanged with cations with +2 or +1 charge. Such substitutions could disrupt the charge balance in the network, leading to changes in the overall structure, connectivity, and stability of the glass. For example, the substitution of a Ti^4+^ cation with a Na^+^ cation may result in a decrease in O–Ti–O crosslinks between borate structural groups and an increased formation of non-bridging oxygens, wherein the smaller Na^+^ network-modifying cations fit more easily into the cages/sites of the existing network, and the Ti^4+^ cations may preferentially assume a network-forming role in forming crosslinks between borate units. A graphical representation illustrating the potential structural modifications induced by the inclusion of network modifiers, such as Na^+^, can be found in previous work [11]. Furthermore, compositional differences between intended and actual concentrations arising from changes during the melting process could potentially contribute to the observed deviations. However, the change in cation charges and the small compositional changes that could arise between intended and actual compositions under normal circumstances are not sufficient to fully explain the scatter in the data. Similar deviations were observed when the B3 content was plotted against the overall cation charge per boron atom based on intended and actual compositions (Figure 7). Evidently, both plots show trendlines far from the predicted slope of −100%/charge and are almost flat. Two possible occurrences that may explain the deviations from expected behavior are as follows: (i) The sample composition changed dramatically from the intended composition, and (ii) phase separation is occurring within the glasses. With regards to the former, one possible cause for differences in the intended versus actual compositions is that the majority of fluorine included in these glasses was lost during glass synthesis. It is possible that BF_3_ formed from the initial components, which is a gaseous compound at room temperature and has a boiling point of −100.3 °C. If BF_3_ burned off during melting, this would result in a decreased concentration of both B and F (and potentially other components) in the samples, thus altering the content of B3 and B4 units in the network. With regards to the latter, phase separation could explain the significant variations observed in the shape and position of the boron signal for 3 and 4-fold coordinated sites in these samples. Specifically, if a non-boron-containing crystal formed, the relative concentration of boron in the remaining glassy matrix would be higher than anticipated. Conversely, if it were a boron-containing crystal, the relative concentration of boron in the glassy phase would be lower than predicted. These differences in boron content throughout the crystal and glassy phases and, consequently, the variations in boron content between predicted and actual concentrations could lead to the fluctuations in %B3 and B4 signals observed in these glasses. 

An additional factor that is likely to contribute to the confounding nature of the borate network structure in these glasses is the inclusion of two intermediate elements, TiO_2_ and ZnO. Interestingly, the coefficients for ZnO and TiO_2_ with respect to %B3 (and %B4) were very similar (e.g., +75.23 * ZnO and +74.86 * TiO_2_), indicating that these constituents had a similar impact on this response, despite their different cationic charges and field strengths. Based on Sun’s single bond strength criterion, Ti and Zn have very similar bond strengths (i.e., 73 and 72 kcals, respectively), indicating that both elements may assume similar roles within the network. Furthermore, these two elements have the highest cationic field strengths of all additive elements in the glasses, indicating that they may dictate structural changes to satisfy their own requirements. For example, the inclusion of TiO_2_ may favor the substitution of B–O–B bonds for B–O–Ti bonds, wherein Ti^4+^ forms ionic crosslinks between borate units. Alternatively, titanium may have disrupted the borate network by forming [TiO_6_]^2−^ octahedrons in which non-bridging oxygens were increasingly formed, and as a result, the fraction of tetrahedrally coordinated boron decreased. The effect of titanium on boron coordination in a titanium- and fluorine-containing borate glass was previously investigated, wherein the increase in TiO_2_ content up to approximately 25 mol% was associated with a decrease in the proportion of tetrahedrally coordinated boron, thus indicating a transformation from B4 to B3 structural units in regions enriched with TiO_2_ [52]. The following Ti coordination change was thought to be the catalyst for this transformation: TiO_4/2_ + 2B(O,F)_4_ → Ti(O,F)_6_ + 2BO_3_. FTIR analysis findings suggested that this coordination change was associated with an increased presence of network-forming TiO_4_ units at low concentrations of TiO_2_ (up to ~10 mol% TiO_2_) and network modifying TiO_6_^2−^ units at higher concentrations of TiO_2_ (~13 to 20 mol% TiO_2_). In addition, similar findings were reported by Lakshmi and Cole [53] in a separate titanium and fluorine-containing borate glass. Specifically, FTIR analysis revealed that with increasing TiO_2_ content (up to 0.7 mol%), the intensity of the band assigned to TiO_6_ octahedral units increased, while the reverse trend ensued for the band assigned to TiO_4_ tetrahedral units. Similarly, with increasing TiO_2_ content, the intensity of the band assigned to BO_3_ units grew at the expense of the BO_4_ unit band. Based on these findings, the authors suggested that titanium assumed the role of a network modifier in the glasses. 

With respect to the role Zn has in modulating borate glass structure, it too can enter the glass network in various coordination states, thus altering the network differently depending on its structure. When functioning as a network modifier, Zn^2+^ ions disrupt B–O–B bonds, thus decreasing the number of bridging oxygens in the network. Conversely, when zinc assumes a network former role, it can take the form of ZnO_4_ structural units, wherein zinc is covalently bonded with four oxygen ions [43]. In a borate glass, the addition of ZnO up to 35 mol% was shown to decrease the boron coordination number as ZnO_4_ units replaced boron atoms in the network [44]. Similarly, for binary zinc–borate glasses, Topper et al. [34] reported that with the addition of ZnO, there was an increasing population of high field strength tetrahedral Zn^2+^ units, which led to the depolymerization of larger borate arrangements due to the increasing number of high charge density borate anions. The authors noted, however, that the increased number of strong Zn^4^–O–B crosslinks partially offset the disruption of the B–O–B bonds. Furthermore, in a separate study on zinc-containing borate glasses, Cetinkaya et al. found that in samples containing greater than 5 wt. % ZnO, zinc acted as a network former. This finding was based on the presence of bands belonging to ZnO_4_ structural units in samples containing greater than 5% ZnO and the absence of these bands otherwise. In addition, the relative intensities of peaks corresponding to bridging oxygens were lower for sample BZ5 (5 wt.% ZnO) and increased for samples BZ10 and BZ15 (10 and 15 wt.% ZnO), thus further supporting the above-mentioned findings. 

Based on the coefficients of the coded equations generated from ^11^B MAS NMR analysis, one could assume that, directionally, both TiO_2_ and ZnO are primarily contributing to an increase in %B3. However, this relationship is not so straight forward, as there are no strong correlations that exist between either of these constituents and boron coordination, and the 3D surface plots show variable responses depending on the relative amounts of the other components. Despite gaining directional information on the influence of these TIIs, the impact of both intermediate elements on boron coordination is somewhat unclear, and further studies may be necessary to ascertain the complex structural relationships that exist in a multi-component borate glass network containing two intermediate elements. 

The strongest correlation observed for the boron coordination was an increased concentration in %B4 (and a decrease in %B3) with increasing CaF_2_ content (Figure 5A). This is evidenced by the 3D surface plot for the B3:B4 ratio (Figure 5B), wherein the ratio is larger where component B:CaF_2_ is at a minimum. According to the coded equation for %B4 (Table 5), CaF_2_ also had the largest individual impact on increasing the response. These findings collectively suggest that CaF2 plays a significant role in modulating the borate network structure. However, the way this constituent affects the network is complex, owing to the presence of both fluorine and calcium in the starting reagent. In the context of fluorine, it has been demonstrated, through NMR analysis of aluminosilicate glasses, that fluorine ions can replace bridging oxygens around silicate tetrahedral units, thereby creating non-bridging, dangling bonds and causing a significant reduction in the glass transition temperature [46]. Similarly, in the borate glass literature, fluorine ions have been shown to substitute the oxygen around trigonal boron atoms due to the similarity in ionic radii between oxygen and fluorine [37]. This substitution results in the formation of non-bridging oxygens and thus increases the fraction of B3 units, contrary to what was observed in this glass system. Alternatively, additional studies have demonstrated fluorine’s preference for forming bonds with other modifier elements present in the network, wherein the replacement of bridging oxygens by nonbridging fluorine ions was not detected [54]. While the mechanism by which fluorine may have induced the increased formation of B4 units in these glasses is not entirely clear, it is important to consider the possible discrepancies between the intended and actual fluorine content in these glasses. As previously discussed, and evidenced through ^19^F MAS NMR analysis, there was an absence of fluorine signal in the samples, indicating that the majority of fluorine may have been burned off in the melting process in gaseous BF_3_ form. If this were in fact the case, it is possible that the correlation between %B3 and CaF_2_ was primarily a result of the calcium. In this regard, Ca^2+^ ions may have increased the conversion of boron from a 3-coordinated state to a 4-coordinated state by fitting into the interstices of the borate network and balancing the negative charges on neighboring BO_4_^−^ groups [16]. As a result of this interaction, the network’s connectivity and viscosity increase. Furthermore, in binary borate glass networks, the addition of CaO up to a maximum of approximately 40 mol% has been shown to increase the glass network connectivity and increase the formation of BO_4_^−^ tetrahedral units at the expense of BO_3_ units, whereafter the reverse trend ensues [55,56]. 

In conjunction with density, XRD, and NMR analyses, the thermal characteristics of the glass networks were also assessed. When comparing the relative effects of each glass constituent on the glass transition temperature of the materials, the individual term with the largest coefficient, and thus the greatest impact on T_g_, was Na_2_CO_3_ (Table 5). This coefficient is positive, indicating that Na_2_CO_3_ contributed to an increase in T_g_. However, to understand the overall impact of Na_2_CO_3_ on T_g_, the interaction terms must be considered. Interestingly, the coefficients corresponding to interaction terms containing Na_2_CO_3_ were all negative, indicating that Na_2_CO_3_ in combination with the other constituents had a relatively greater negative impact on the glass transition temperature (i.e., contributed to a decrease in T_g_). This was further supported by the strong correlation observed between T_g_ and Na_2_CO_3_ content, wherein there was a decreased T_g_ value with increasing Na_2_CO_3_ content (Figure 8B). Furthermore, this effect was verified by adjusting the concentration of component E: Na_2_CO_3_ in the 3D surface plot, wherein the surface clearly shifted to higher temperatures at the lowest Na_2_CO_3_ content and vice versa. These combined findings demonstrate the complex role that sodium may have in these networks and how that role may differ when in combination with other constituents. This is a great example of why it is crucial to understand not only the individual effects of glass constituents, but also the complex, and evidently influential, synergistic effects that may occur between them. With regards to the mechanism by which Na_2_CO_3_ may cause a decrease in glass transition behavior, sodium has previously been used as an effective flux in glass materials, thus increasing the ease of melting and homogenization processes [57]. This is because, past a certain concentration, the introduction of electropositive cations of alkali metals, such as Na^+^, is known to break up the interlinked borate network by converting bridging oxygen atoms into non-bridging oxygen ions [58]. This conversion decreases the network connectivity of the glass and thus alters its thermal characteristics, such as viscosity and glass transition temperature. However, based on the principles of the borate anomaly in binary Na_2_O–B_2_O_3_ glasses, increasing the sodium content initially induces the formation of BO_4_^−^ tetrahedral units at the expense of BO_3_ units until the fraction of modifier hits a maximum, whereupon non-bridging oxygens begin to form [11]. Because (1) the glasses under investigation contain modifier fractions ranging from 30–55 mol% that include variable concentrations of alkaline, alkaline–earth, transition metal, and halogen elements, and (2) there were no evident correlations between Na_2_CO_3_ content and %B4 and %B3, it is difficult to ascertain the role of Na_2_CO_3_ in modulating B3 and B4 fractions and how that might affect the thermal properties of these glasses. Although the boron coordination is often assumed to be correlated to the glass transition temperature [59], there was no correlation between the glass transition temperature and %B3 or %B4 in these glasses. These results indicate that the mechanism by which Na_2_CO_3_ caused a decrease in T_g_ was not related to the relative fractions of B3 and B4 units in the network. As such, an alternative theory for the decreased T_g_ values is that, upon increasing the Na_2_CO_3_ content, B–O bonds are increasingly substituted by lower bond dissociation energy Na–O bonds in the network. For instance, the addition of Na_2_O up to 50 mol% has previously been shown to decrease the glass transition temperature in Na_2_O–ZnO–B_2_O_3_ glasses, wherein authors attributed this decrease to the low bond association energy of Na–O bonds (257 kJ/mol) compared to B–O bonds (806 kJ/mol) [60]. 

Among the many benefits of employing predictive modelling approaches in the design and development of novel glass materials, perhaps one of the most impactful is the ability to predict and optimize new formulations tailored to specific properties. Optimization studies offer a systematic and strategic approach to enhancing the functionality and performance of novel materials. These studies allow researchers to fine-tune compositions with precision, ensuring that the material properties align optimally with desired outcomes. By leveraging surface regression methodologies, we can not only navigate the complexities of multidimensional systems but also discover optimal solutions within vast compositional spaces using limited time and resources. By identifying the most favorable compositions and configurations, these studies streamline the materials discovery process, paving the way for quicker translation of novel materials into practical applications. In the current investigation, the optimization and validation study was used to assess the predictive capabilities of the models by comparing the measured properties of optimized solutions against the predicted values of the model. As shown in Table 8 and Table 9, the actual values derived from density and DSC analyses of the two optimized glasses are within the 95% prediction interval, confirming that the density and T_g_ models are predicting accurately at both optimized settings. However, the results arising from ^11^B MAS NMR analysis fell outside the model’s 95% prediction interval, indicating that the %B3 and %B4 models were not predicting as accurately as desired. Although the models generated for %B3 and %B4 and B3:B4 were statistically significant, the ^11^B MAS NMR results (specifically the observed data scattering and deviation from expected behavior) suggested that the changes in borate network structure were difficult to accurately predict in these systems. This was somewhat expected given the unpredictable nature of the borate glass structure within the anomalous region. In light of these findings, it is worth noting that the measured values for %B3 and %B4 were provided with a ±2 to account for measurement error/variability in chemical shift. If the ±2 error is applied to the actual values pertaining to %B3 and %B4, there is a scenario in which these values fall within the 95% prediction interval for both optimized glasses. An additional consideration is that the optimization study was limited by conducting only one confirmation run to confirm the accuracy of the models. In future work, both optimized formulations should be synthesized and characterized at least three times to account for any possible variations in glass synthesis, measurement error, or human error. Ultimately, the results of the current optimization study demonstrate that by employing a predictive modelling approach, it was possible to accurately predict the individual and interaction effects of various glass constituents on the density and thermal properties of these complex, multi-component glasses. Although the optimization and validation results suggested that the models generated from ^11^B MAS NMR analysis were not predicting as accurately as desired, the models still provide directional information on the individual and interaction effects of each constituent on the borate network structure.

## 5. Conclusions

The application of a predictive modelling approach, based on Design of Mixtures (DoM), has successfully elucidated the individual and interaction effects of B_2_O_3_, CaF_2_, TiO_2_, ZnO, and Na_2_CO_3_ on the structure and properties of oxyhalide borate glasses with modifier fractions ≥ 30 mol%. This study highlights the non-linear and complex relationships that exist within multi-component glass systems, particularly within the anomalous region of borate glasses. Subsequent to developing statistically significant models for various material characteristics, the authors illustrated how to predict and optimize new glass compositions tailored for specific properties, offering new insights into the design and development of soluble glass networks. Although some challenges in predicting changes in the borate network structure are acknowledged, the study demonstrates the effectiveness of systematic predictive modelling in overcoming traditional barriers in glass science. Such advancements will open new avenues for the accelerated discovery and development of novel glasses for a wide range of applications. Future work will focus on further elucidating the observed phenomena, particularly the scattering in ^11^B MAS NMR data and the potential loss of fluorine during synthesis, to refine our predictive capabilities and expand our understanding of these complex materials.

## Figures and Tables

**Table 1:** Mixture components and design constraint summary.

Component	Units	Minimum [mol%]	Maximum [mol%]	Coded Low	Coded High	Mean	Std. Dev.
B_2_O_3_	Mol%	45	70	+0 ↔ 45	+0.55 ↔ 70	59.69	7.65
CaF_2_	Mol%	0	20	+0 ↔ 0	+0.44 ↔ 20	11.70	7.08
TiO_2_	Mol%	0	10	+0 ↔ 0	+0.22 ↔ 10	5.32	3.90
ZnO	Mol%	5	20	+0 ↔ 5	+0.33 ↔ 20	13.28	5.78
Na_2_CO_3_	Mol%	5	15	+0 ↔ 5	+0.22 ↔ 15	10.01	4.06

**Table 2:** Compositions of the 23 glasses (mol%), as identified by the constraints outlined in Table 1. Within the table there are four replicate compositions consisting of 1 and 12, 3 and 6, 4 and 5, and 17 and 21.

	B_2_O_3_	CaF_2_	TiO_2_	ZnO	Na_2_CO_3_
1	70.00	13.47	3.10	5.00	8.43
2	45.00	20.00	10.00	20.00	5.00
3	54.61	11.44	10.00	13.33	10.62
4	63.26	2.08	4.76	20.00	9.90
5	63.26	2.08	4.76	20.00	9.90
6	54.61	11.44	10.00	13.33	10.62
7	64.78	6.38	8.83	5.00	15.00
8	64.75	8.65	6.16	15.43	5.00
9	45.00	20.00	7.77	12.23	15.00
10	70.00	0.00	10.00	12.75	7.25
11	55.24	13.99	5.78	19.99	5.00
12	70.00	13.47	3.10	5.00	8.43
13	50.44	14.34	0.22	20.00	15.00
14	68.01	3.17	1.36	12.46	15.00
15	60.00	20.00	0.00	5.00	15.00
16	53.20	17.96	9.74	5.00	14.10
17	56.49	20.00	2.01	13.54	7.97
18	60.00	20.00	10.00	5.00	5.00
19	55.00	0.00	10.00	20.00	15.00
20	69.82	6.73	0.00	18.44	5.00
21	56.49	20.00	2.01	13.54	7.97
22	65.89	16.23	0.00	12.88	5.00
23	57.01	7.56	2.86	17.57	15.00

**Table 3:** Criteria for optimized glass formulations.

Optimization Criteria						
Factor/Response	Units	Lower Limit	Upper Limit	Importance	Goal: Optimized Glass A	Goal: Optimized Glass B
B_2_O_3_	Mol%	45	70	3	Maximize	In range
CaF_2_	Mol%	0	20	3	In range	In range
TiO_2_	Mol%	0	10	3	In range	In range
ZnO	Mol%	5	20	3	In range	In range
Na_2_CO_3_	Mol%	5	15	3	In range	In range
Density	g/cm^3^	2.4	3.1	5	Target = 2.5	Target = 2.9
%B4	%	23.59	42.41	3	Minimize	Maximize
T_g_	°C	471.6	563.8	3	Target = 540	Target = 510
Crystallinity	%	0.86	19.62	3	Minimize	Minimize

**Table 4:** Material characterization results for particle size, density, crystallinity, glass transition temperature, and boron structural units for each glass composition.

	Dx(10) [μm]	Dx(50) [μm]	Dx(90) [μm]	Density [g/cm^3^]	% Crystallinity [%]	T_g_ [°C]	%B3	%B4	B3:B4
1	120	332	731	2.50	1.7	537.5	66.3	33.6	1.97
2	114	278	605	3.17	1.7	532	66.1	33.9	1.95
3	145	372	755	2.85	3.1	518.8	64.7	35.3	1.83
4	128	319	658	2.73	3.5	503.6	69.2	30.7	2.25
5	104	259	593	2.73	3.8	517	69.5	30.5	2.28
6	111	268	565	2.84	3.2	516.6	64.3	35.7	1.80
7	113	299	638	2.48	12	494.1	66.4	33.5	1.98
8	122	329	676	2.63	3.7	545.6	69.3	30.7	2.25
9	119	302	626	2.83	1.5	471.6	63.9	36.1	1.77
10	154	380	742	2.43	16.9	487.2	76.4	23.6	3.24
11	130	337	677	2.94	2.7	551	64.3	35.7	1.80
12	111	291	647	2.47	2.2	532.6	65.9	34.1	1.93
13	118	319	697	2.90	1.9	482.1	63.8	36.2	1.76
14	151	407	769	2.57	1.8	499.5	66.7	33.3	2.00
15	135	367	725	2.64	2.7	516.6	57.6	42.4	1.36
16	104	261	574	2.77	3.2	504.7	61.0	39.0	1.57
17	134	334	666	2.88	1.9	527.6	61.4	38.6	1.59
18	147	373	734	2.68	2.8	563.6	63.4	36.6	1.73
19	124	302	589	2.80	15	490.9	67.2	32.8	2.05
20	120	334	656	2.63	2.4	541.3	70.4	29.5	2.38
21	98.4	218	458	2.81	2.1	535.3	61.4	38.6	1.59
22	132	330	658	2.66	1.8	563.8	64.0	36.0	1.78
23	136	377	728	2.74	2.3	495.1	64.0	36.0	1.78

**Table 5:** Regression model coded equations based on L_Pseudo components for each response generated through Design-Expert. The summarized ANOVA including the R^2^, adjusted R^2^, predicted R^2^, C.V. (%), and adequate precision is also included. The predicted R^2^ for each response is within reasonable agreement to the adjusted R^2^ (i.e., difference of <0.2) and the adequate precision values are greater than 4.

Response	Regression Model	R^2^	Adjusted R^2^	Predicted R^2^	C.V. (%)	Adequate Precision
Density	1.87 * B_2_O_3_ + 2.88 * CaF_2_ + 5.05 * TiO_2_ + 3.91 * ZnO + −0.66 * Na_2_CO_3_ + 0.73 * B_2_O_3_CaF_2_ + −3.33 * B_2_O_3_TiO_2_ + −0.21 * B_2_O_3_ZnO + 5.68 * B_2_O_3_Na_2_CO_3_ + −2.01 * CaF_2_TiO_2_ + −0.53 * CaF_2_ZnO + 3.66 * CaF_2_Na_2_CO_3_ + −3.46 * TiO_2_ZnO + 1.07 * TiO_2_Na_2_CO_3_ + 2.70 * ZnONa_2_CO_3_	0.99	**0.98**	**0.83**	0.92	36.69
% Crystallinity	10.08 * B_2_O_3_ + 34.51 * CaF_2_ + 115.20 * TiO_2_ + 24.56 * ZnO + 114.15 * Na_2_CO_3_ + −64.99 * B_2_O_3_CaF_2_ + −50.99 * B_2_O_3_TiO_2_ + −51.33 * B_2_O_3_ZnO + −157.37 * B_2_O_3_Na_2_CO_3_ + −284.31 * CaF_2_TiO_2_ + −57.53 * CaF_2_ZnO + −203.37 * CaF_2_Na_2_CO_3_ + −145.93 * TiO_2_ZnO + −159.41 * TiO_2_Na_2_CO_3_ + −186.40 * ZnONa_2_CO_3_	0.99	**0.97**	**0.85**	17.08	27.01
T_g_	442.42 * B_2_O_3_ + 461.74 * CaF_2_ + 569.54 * TiO_2_ + 525.10 * ZnO + 673.09 * Na_2_CO_3_ + 489.77 * B_2_O_3_CaF_2_ + −115.04 * B_2_O_3_TiO_2_ + 194.12 * B_2_O_3_ZnO + −161.92 * B_2_O_3_Na_2_CO_3_ + 200.86 * CaF_2_TiO_2_ + 10.17 * CaF_2_ZnO + −467.98 * CaF_2_Na_2_CO_3_ + 65.39 * TiO_2_ZnO + −535.48 * TiO_2_Na_2_CO_3_ + −509.61 * ZnONa_2_CO_3_	0.99	**0.97**	**0.91**	0.92	24.14
%B3	94.53 * B_2_O_3_ + 60.04 * CaF_2_ + 74.86 * TiO_2_ + 75.23 * ZnO + 52.91 * Na_2_CO_3_ + −65.06 * B_2_O_3_CaF_2_ + −12.24 * B_2_O_3_TiO_2_ + −46.87 * B_2_O_3_ZnO + −54.87 * B_2_O_3_Na_2_CO_3_ + −9.13 * CaF_2_TiO_2_ + −0.91 * CaF_2_ZnO + 13.00 * CaF_2_Na_2_CO_3_ + −20.20 * TiO_2_ZnO + −0.19 * TiO_2_Na_2_CO_3_ + 15.02 * ZnONa_2_CO_3_	0.99	**0.99**	**0.93**	0.59	60.79
%B4	5.46 * B_2_O_3_ + 39.96 * CaF_2_ + 25.14 * TiO_2_ + 24.77 * ZnO + 47.08 * Na_2_CO_3_ + 65.06 * B_2_O_3_CaF_2_ + 12.23 * B_2_O_3_TiO_2_ + 46.87 * B_2_O_3_ZnO + 54.87 * B_2_O_3_Na_2_CO_3_ + 9.13 * CaF_2_TiO_2_ + 0.91 * CaF_2_ZnO + −13.00 * CaF_2_Na_2_CO_3_ + 20.20 * TiO_2_ZnO + 0.19 * TiO_2_Na_2_CO_3_ + −15.02 * ZnONa_2_CO_3_	0.99	**0.99**	**0.93**	1.13	60.79
B3:B4	4.65 * B_2_O_3_ + 2.24 * CaF_2_ + 6.26 * TiO_2_ + 2.44 * ZnO + 0.43 * Na_2_CO_3_ + −7.67 * B_2_O_3_CaF_2_ + −3.86 * B_2_O_3_TiO_2_ + −3.51 * B_2_O_3_ZnO + −4.85 * B_2_O_3_Na_2_CO_3_ + −7.82 * CaF_2_TiO_2_ + −0.82 * CaF_2_ZnO + 2.17 * CaF_2_Na_2_CO_3_ + −5.17 * TiO_2_ZnO + −6.06 * TiO_2_Na_2_CO_3_ + 1.31 * ZnONa_2_CO_3_	0.99	**0.99**	**0.89**	2.17	54.56

**Table 6:** Identification of crystals precipitated in samples #7, 10, 19.

Sample No.	COD ID	Chemical Name
7	COD 5000223	Titanium oxide—anatase
10	COD 1511015	Ti B0.024 O_2_
	COD 1534781	TiO_2_
19	COD 9004142	Titanium oxide—rutile
	COD 1511015	Ti B0.024 O_2_

**Table 7:** Composition of optimized formulations and predicted values for select parameters.

Glass	B_2_O_3_	CaF_2_	TiO_2_	ZnO	Na_2_CO_3_	Density [g/cm^3^]	%B3	%B4	T_g_ [°C]	Desirability
A	70.0	8.1	3.2	13.7	5.0	2.5	70.7	29.2	539.9	0.902
B	51.9	20.0	3.5	14.8	9.81	2.9	62.2	37.8	510.0	0.942

**Table 8:** Predicted and actual values for density, T_g_, %B3, and %B4 responses for optimized glass A. Actual values that fall within the 95% prediction interval indicate that the model for that given response is predicting accurately at the optimized settings. Actual values that do not fall within the 95% prediction interval are highlighted in red.

Optimized Glass A				
Response	Predicted	95% Prediction Interval Low	Actual	95% Prediction Interval High
Density [g/cm^3^]	2.5	2.428	2.548	2.571
T_g_ [°C]	539.9	526.464	534.2	553.602
B3 [%]	70.75	69.637	69	71.853
B4 [%]	29.25	28.147	31	30.363

**Table 9:** Predicted and actual values for density, T_g_, %B4, and %B3 responses for optimized glass B. Actual values that fall within the 95% prediction interval indicate that the model for that given response is predicting accurately at the optimized settings. Actual values that do not fall within the 95% prediction interval are highlighted in red.

Optimized Glass B				
Response	Predicted	95% Prediction Interval Low	Actual	95% Prediction Interval High
Density [g/cm^3^]	2.9	2.831	2.874	2.971
T_g_ [°C]	510	496.5	511	523.159
B3 [%]	62.19	61.118	60	63.296
B4 [%]	37.81	36.704	40	38.882

## Data Availability

The original contributions presented in the study are included in the article, further inquiries can be directed to the corresponding author.
